# EZH2 Inhibitors Suppress Colorectal Cancer by Regulating Macrophage Polarization in the Tumor Microenvironment

**DOI:** 10.3389/fimmu.2022.857808

**Published:** 2022-04-01

**Authors:** Chen Li, Jiagui Song, Zhengyang Guo, Yueqing Gong, Tengrui Zhang, Jiaqi Huang, Rui Cheng, Xiaotong Yu, Yanfang Li, Li Chen, Xiaojuan Ma, Yan Sun, Yan Wang, Lixiang Xue

**Affiliations:** ^1^ Department of Radiation Oncology, Peking University Third Hospital Cancer Center, Peking University Third Hospital, Beijing, China; ^2^ Center of Basic Medical Research, Institute of Medical Innovation and Research, Peking University Third Hospital, Beijing, China

**Keywords:** EZH2, EZH2 inhibitor, colorectal cancer, macrophage, tumor microenvironment

## Abstract

EZH2 inhibitors (EZH2i), a class of small-molecule inhibitors that target EZH2 to exert anti-tumor functions, have just been approved by the US Food and Drug Administration (FDA) in treatment of adults and adolescents with locally advanced or metastatic epithelioid sarcoma. The application of EZH2i in several solid tumors is still in different stages of clinical trials and needs to be further validated. As a key epigenetic regulator, besides its role in controlling the proliferation of tumor cells, EZH2 has been implicated in the regulation of various immune cells including macrophages. But there are still controversial research results at present. Colorectal cancer (CRC) is a common malignant tumor that highly expresses EZH2, which has the third highest incidence and is the second leading cause of cancer-related death worldwide. Studies have shown that the numbers of M2-type tumor-associated macrophages (TAMs) are highly associated with the progression and metastasis of CRC. In the current study, we aim to investigate how EZH2 modulates the polarization of macrophages in the tumor microenvironment (TME) of CRC, and compare the role of two different EZH2 inhibitors, EPZ6438 and GSK126. We applied a 3D culture method to demonstrate that EZH2i did indeed suppress the proliferation of CRC cells *in vitro*. *In vivo*, we found that the percentage of CD206^+^ macrophages of the TME was decreased under the treatment of EPZ6438, but it increased upon GSK126 treatment. Besides, in the co-culture system of macrophages and CRC cells, EPZ6438 led to significant elevation of M1 markers and reduction of M2 markers. Furthermore, mechanistic studies validated by ChIP-qPCR demonstrated that EZH2i inhibit EZH2-mediated H3K27me3 levels on the promoters of STAT3, an essential transcription factor for M1 macrophage polarization. Therefore, our data suggested that EZH2i not only suppress CRC cell proliferation directly, but also regulate macrophage by skewing M2 into effector M1 macrophage to exert a tumor suppressive effect. Moreover, our study provided new insight for better understanding of the role of two kinds of EZH2i: EPZ6438 and GSK126, which may pave the way in treating CRC by targeting cancer cells and immune cells *via* this epigenetic approach in the future.

## Introduction

Enhancer of zeste homolog 2 (EZH2) is the enzymatic subunit of polycomb repressive complex 2 (PRC2), which can catalyze tri-methylates lysine 27 of histone H3 (H3K27me3) to mediate gene transcriptional silencing and chromatin compaction (1). Various studies have elucidated the complex role of EZH2 in biological processes and cancer-related events (2). Moreover, epigenetic modifications associated with EZH2 contribute to the regulation of subsets of immune cells, including various T cells (3, 4), innate immune cells such as tumor-associated macrophages (TAMs) (5), myeloid-derived suppressor cells (6), NK cells (7), and so on. It is reported that EZH2 regulates inflammatory responses and polarization of macrophages through genetic targeting of EZH2 or pharmacological EZH2 inhibitors (8). However, the role of regulation of EZH2 on macrophage polarization remains controversial.

Tumor-associated macrophages are a group of highly plastic cells in the tumor microenvironment that are characterized by their plasticity and heterogeneity (9). There are two main TAM subtypes *in vivo*, including classically activated macrophages (M1) and alternatively activated macrophages (M2) (10). M1 macrophages (CD86^+^ and CD80^+^) release pro-inflammatory cytokines (e.g., IL-6, IL-12, TNF-α) to play an anti-tumor role, while M2 macrophages (CD206^+^) release pro-tumor cytokines (e.g., IL-4, IL-10, IL-13) to promote tumor progression and metastasis (11). Although some studies have investigated the role of EZH2 on macrophages polarization, the conclusion varies in different settings, i.e., suppression of EZH2 remodeled macrophages toward pro-inflammatory M1 inhibiting tumor proliferation in glioma (5, 12). However, in experimental model of autoimmune diseases including dextran sulfate sodium (DSS)-induced colitis and experimental autoimmune encephalomyelitis (EAE), EZH2 in the macrophage induced autoimmune disease progression, and inhibition of EZH2 reduced pro-inflammatory responses (13).

Colorectal cancer (CRC) is one of the most frequent malignant tumors (14). Particularly, accumulated data verified that expression levels of EZH2 are positively correlated with CRC grades and negatively correlated with survival, and EZH2 inhibitors have been demonstrated to have therapeutic effects in CRC treatment (15). In addition, macrophages are the most common non-neoplastic cells in the CRC microenvironment, and their polarization state has prognostic significance in CRC (16). However, it is not clear how EZH2 and its selective inhibitors regulate the polarization and inflammatory responses of macrophages in the colorectal tumor microenvironment.

Among all EZH2 inhibitors (EZH2i), tazemetostat (EPZ6438) has been used for the treatment of epithelioid sarcomas that cannot be removed by surgery, becoming the first EZH2i approved by the US Food and Drug Administration (FDA) in 2020 (17, 18). But the clinical trial outcomes of GSK126, another EZH2 inhibitor, in patients with diffuse large B-cell lymphoma (DLBCL), follicular lymphoma (FL), multiple myeloma (MM), and other solid tumors were unsatisfactory (19, 20). Huang et al. partly explained why GSK126 failed in the clinical trial, as they found that GSK126 drove myeloid-derived suppressor cells (MDSCs) to suppress anti-tumor immunity (6). At present, there are few studies comparing these different drugs on the effects of innate immunity on tumor growth.

In this study, we aimed to investigate the regulation of EZH2 on macrophage polarization in the tumor microenvironment by applying both a co-culturing system and tumor-bearing mouse model. Due to the different performances of EPZ6438 and GSK126 in clinical trials, we further explored the different effects of these two inhibitors on CRC simultaneously.

## Materials and Methods

### Mice and Tumor Models

Female C57BL/6 mice (6-8 weeks) were purchased from Beijing Vital River Laboratory (Beijing, China). All animal protocols were approved by the Animal Care and Use Committee of Peking University and all mice were housed in specific-pathogen-free (SPF) conditions. For *in vivo* studies, GSK126 and EPZ6438 (Cat^#^: S7061 and S7128, Selleck) or vehicle was administered intraperitoneally at a dose of 50 mg/kg daily and 100 mg/kg every three days, respectively. MC38 cells (5×10^5^) were implanted subcutaneously in female C57BL/mice (n = 5 mice/group). All treatment was initiated approximately 4-5 days after implantation and tumor growth was measured using calipers every 2 days. Tumor volume was calculated as follows: V =(0.5 × length ×width ×width). Tumors were harvested and isolated into single cells from euthanized mice at the indicated time points using a tumor dissociation kit-mouse (Cat^#^: 130-096-730, Miltenyi), and immune cells were analyzed by flow cytometry.

### Depletion of Macrophages

The mice were administrated clodronate liposomes (CL) to deplete macrophages *in vivo*, as reported previously (21). To determine the efficiency of macrophage depletion, mice were injected intraperitoneally (i.p.) with CL (12.5 mg/kg, Cat#: 40337ES08, Yeasen) and PBS, respectively, to evaluate the deplete efficiency of spleen macrophages at 1 and 3 days by flow cytometry analysis. For the tumor-bearing mouse model, mice were administrated with CL and PBS 1 day before tumor bearing, and treatment of CL and PBS was given every 3 days during the subsequent experiment.

### Cell Lines and Cell Culture

MC38 and RAW264.7 cells were obtained from the American Type Culture Collection (ATCC). MC38 was cultured in basic DMEM supplemented with 10% fetal bovine serum (FBS), 1% penicillin/streptomycin (P/S), 1% NEAA, 1% sodium pyruvate, 1% HEPES, and 1% glutamine. RAW264.7 was cultured in basic DMEM supplemented with 10% FBS and 1% P/S. All cell lines were mycoplasma-negative and used within 10 passages.

### 3D Spheroid Culture

MC38 and RKO tumor spheroids were created using IBAC SR1 3D plates, as reported previously (21). Briefly, 10,000 MC38 or RKO cells were mixed with Matrigel (Cat#: 356231, Corning) at a 1:1 ratio and seeded into IBAC SR1 3D plates (Cat#: SR109610, Daxiang Biotech). Spheroids were grown for 6 days for formation and treated with indicated concentrations of GSK126 for 48 h after spheroid formation. DMSO was used as the solvent control.

### Cell Viability Assay

RAW264.7 cells were seeded in 96-well plates at a density of 1.5 × 10^4^ cells/100 µl overnight and treated with GSK126 and EPZ6438 at different concentration gradients for 48 h. A CCK8 assay (Cat^#^: CK04, Dojindo Laboratories) was then carried out with a 10 ul sample and incubated at 37°C for 3 h. The absorbance (optical density, OD) was read at a wavelength of 450 nm on an ELISA plate reader.

Viability of 3D tumor spheroids was analyzed using a 3D Cell-titer Glo (Cat^#^: G9681, Promega) reagent according to the manufacturer’s instructions, and luminescence was measured on a GloMax 96 Microplate luminometer.

### Cell Proliferation Assay

RAW264.7 cells and MC38 cells were seeded in 96-well plates at a density of 1.5 × 10^4^ cells/100 ul and 1 × 10^4^ cells/100 µl overnight, respectively. The cells were replaced with fresh medium containing different concentrations of GSK126 and EPZ6438. Then, the real-time proliferation of those cells was detected every 4 h until the end of 48 h by the Incucyte^®^ live cell analysis system. The phase confluence normalized to 0 h of cells was analyzed by Basic Analyzer processing modules. Mean values based on 3 × 3 wells per condition and standard error were collated.

### Bone Marrow-Derived Macrophages (BMDMs) Culture

Femora and tibiae of C57BL/6 mice were harvested and the bone marrow cells from all bones were flushed out. Then after centrifuging cells for 5 min at 500 ×g, erythrocytes were eliminated using red blood cell lysing buffer (Cat^#^: R1010, Solarbio). The remaining cells were seeded in plates and incubated in complete DMEM medium overnight, and then replaced with fresh medium with 10 ng/ml of recombinant mouse M-CSF (Cat^#^: AF-315-02, PeproTech), for 2 days.

### Cell Treatment

EPZ6438 and GSK126 were dissolved in dimethyl sulfoxide (DMSO) for the treatment of cells. The final concentration of DMSO was less than 0.1% (v/v). To evaluate the direct effect of EZH2i on RAW264.7 and MC38 cells, cells were treated with EPZ6438 and GSK126 for 48 h after adherent 12 h. To evaluate the effect of EZH2i on RAW264.7 cells in tumor-condition medium, macrophages were treated with tumor medium for 24 h, then given EZH2i treatment for another 48 h. To evaluate the effect of EZH2i on M2-polarized macrophages, RAW264.7 cells were pretreated with IL-4 (20 ng/ml, Cat^#^: 214-14-20, PeproTech) for 24 h, then given EZH2i for another 48 h with or without IL-4 stimulation. Control cells were kept in culture with DMSO for the entire experimental period.

### Macrophage Polarization

The original RAW264.7 cell line represents M0 macrophages. To obtain M1-polarized macrophages, RAW264.7 cells were treated with lipopolysaccharide (LPS) (100 ng/ml, Cat^#^: L2880, Sigma Aldrich) for 12 h. To generate M2-polarized macrophages, RAW264.7 cells were treated with IL-4 (20 ng/ml) for 24 h.

### Conditioned Tumor Medium Preparation

MC38 tumor cells were plated in 100 mm dishes in basic DMEM supplemented with 10% fetal bovine serum (FBS), 1% penicillin/streptomycin (P/S), 1% NEAA, 1% sodium pyruvate, 1% HEPES, and 1% glutamine. At 80-90% cell confluence, the medium was collected and centrifuged at 2000 rpm, RT, for 10 min. The supernatant was retained and stored at -20°C in 50 ml tubes until use.

### Flow Cytometry

For the mouse model, tumors were harvested and isolated into single cells using a tumor dissociation kit-mouse and supporting machine GentleMACS™ (^©^MiltenyiBiotec) and then filtered twice with 300 mesh screens. Cells were then stained with antibodies against CD45-BV510, CD11b-FITC, F4/80-PE, CD206-BV421, and CD86-APC for 30 min at 4°C in the dark and 7-AAD was added for the last 5 min. Then, the cells were washed twice and resuspended in 100 μL of phosphate-buffered saline (PBS). For the cell line experiment, RAW264.7 cells or BMDMs were stained with antibodies against CD11b-FITC, F4/80-PE, CD86-BV605, and Zombie NIR™ for live and dead cells according to the standard cell staining protocol (all antibodies were from BioLegend). The cells were analyzed by a CytoFLEX S (Beckman coulter) and re-analyzed using the Kaluza software (Beckman coulter).

### ChIP-qPCR

MC38 cells plated in 10 cm dishes were treated with the methods shown in **Figure 3A**. Then cells were fixed with 1% formaldehyde for 10 min at room temperature. To stop the reaction, glycine was added to a final concentration of 0.125 M at room temperature for 5 min. Cells were scraped into cold PBS with proteinase inhibitor and transferred with contents of each group to a 15 mL tube. A ChIP assay was performed using a Simple ChIP Plus Enzymatic Chromatin IP Kit (Magnetic Beads) (Cat^#^: 9005, Cell Signaling Technology) and anti-histone H3K27me3 (Cat^#^: 9733, Cell Signaling Technology) according to the procedures provided by the manufacturer. The final ChIP DNA samples were then used as templates in qPCR reactions. Primers were designed upon different promoter regions shown in **Figure 5E**.

### Real-Time PCR Assay (RT-PCR)

Total RNA extracted from cells was isolated using Trizol (Cat^#^: 15596018, Thermo Scientific), and RNA concentration and purity were measured using Nanodrop 2000. cDNA was synthesized from 1 ug of total RNA using the Hifair^®^ III 1st Strand cDNA Synthesis SuperMix for qPCR (gDNA digester plus) (Cat^#^: 11139ES, Yeasen). The sequences of the primers (Tsingke) used for RT-PCR are listed in **Table 1**. Quantitative RT-PCR was carried out in a 96-well plate using SuperReal PreMix Plus reagents (Cat^#^: FP205-03, TIANGEN) on Bio-rad CFX manager according to the manufacturer’s protocol, and all results were processed by the double-delta method (2−ΔΔCt).

**Table 1:** Primer sequences.

	Primer	sequence (5'-3')
1	Forward primer mouse CD86	AACTTACGGAAGCACCCACG
2	Reverse primer mouse CD86	CTCCACGGAAACAGCATCTGAG
3	Forward primer mouse iNOS	CGAAACGCTTCACTTCCAA
4	Reverse primer mouse iNOS	TGAGCCTATATTGCTGTGGCT
5	Forward primer mouse TNFα	GAGTGACAAGCCTGTAGCC
6	Reverse primer mouse TNFα	CTCCTGGTATGAGATAGCAAA
7	Forward primer mouse CD206	TTTGGAGGCTGATTACGAGCA
8	Reverse primer mouse CD206	TGGTTCACCGTAAGCCCAAT
9	Forward primer mouse MRC1	AAGGCTATCCTGGTGGAAGAA
10	Reverse primer mouse MRC1	AGGGAAGGGTCAGTCTGTGTT
11	Forward primer mouse Arg1	AACACGGCAGTGGCTTTAACC
12	Reverse primer mouse Arg1	GGTTTTCATGTGGCGCATTC
13	Forward primer mouse IL-1b	TGCCACCTTTTGACAGTGATG
14	Reverse primer mouse IL-1b	TGATGTGCTGCTGCGAGATT
15	Forward primer mouse IL-18	GACAGCCTGTGTTCGAGGAT
16	Reverse primer mouse IL-18	GGTGGATCCATTTCCTCAAAGG
17	Forward primer mouse GSDME	GCGCACTAGCAGAAATGCC
18	Reverse primer mouse GSDME	CAGAGGCAAACAATCGCTGC
19	Forward primer mouse GSDMD	GCAGAGGCGATCTCATTCCG
20	Reverse primer mouse GSDMD	CCAAAACACTCCGGTTCTGGTT
21	Forward primer mouse GAPDH	TTCACCACCATGGAGAAGGC
22	Reverse primer mouse GAPDH	GGCATGGACTGTGGTCATGA

### Multiplexed Immunofluorescence (m-IF)

An Opal seven-color kit (Cat^#^: NEL811001KT, Akoya Bioscience) was used for m-IF. Five-micrometer formalin-fixed, paraffin-embedded (FFPE) tumor sections of the mouse model were stained using a reference protocol (22) and the panel contained antibodies against DAPI (Cat^#^: 44010A, BestBio), CD206 (1:500, dye 620, Cat^#^: 24595, Cell Signaling Technology), CD86 (1:500, dye 690, Cat^#^: 19589, Cell Signaling Technology), and F4/80 (1:500, dye 540, Cat^#^:70076, Cell Signaling Technology). Slides were imaged using a Vectra Polaris automated multispectral microscope (Akoya Bioscience). Whole slide scans were performed using the ×20 objective lens. Several regions of interest (ROIs) were selected with fixed-size stamps in Phenochart (PerkinElmer), based on the previously acquired whole slide scan images of tumors. Acquired images were analyzed by inForm tissue finder software (Akoya bioscience).

### Western Blot Analysis

Whole-cell lysates were prepared using RIPA buffer with protease inhibitors. After protein quantification (Pierce BCA Protein Assay Kit, Thermo scientific, USA), 20 μg of lysate was separated by electrophoresis on SDS-PAGE gels and blotted onto a polyvinylidene fluoride (PVDF) membrane (Cat^#^: ISEQ00010, merck milipore). Membranes were incubated with antibodies against EZH2 (Cat^#^: 5246S, Cell Signaling Technology), Arg1 (Cat^#^: 93668T, Cell Signaling Technology), iNOS (Cat^#^: MAB9502-SP, R&D system), or GADPH (Cat^#^: C1312, Applygen) overnight at 4°C. Then, the membranes were incubated with anti-rabbit/mouse IgG and HRP-linked antibody (Cat^#^: ZB-2305, ZSGB-Bio) for 1 h at room temperature. After washing twice for 10 min with TBST, the membranes were exposed to obtain the blot images with ECL Reagent (Cat^#^: P1030-100, Applygen).

### Statistical Analysis

All statistical analyses were performed using GraphPad Prism 8 software. The differences between two groups were analyzed by Student’s t-test. The differences between more than two groups were analyzed by one-way analysis of variance (ANOVA) with a multiple comparison post-test. P < 0.05 meant a statistically significant difference.

## Results

### EPZ6438 and GSK126 Had Different Effects on Colorectal Tumor Proliferation and Growth *In Vivo* and *In Vitro*

**Figure 1:**
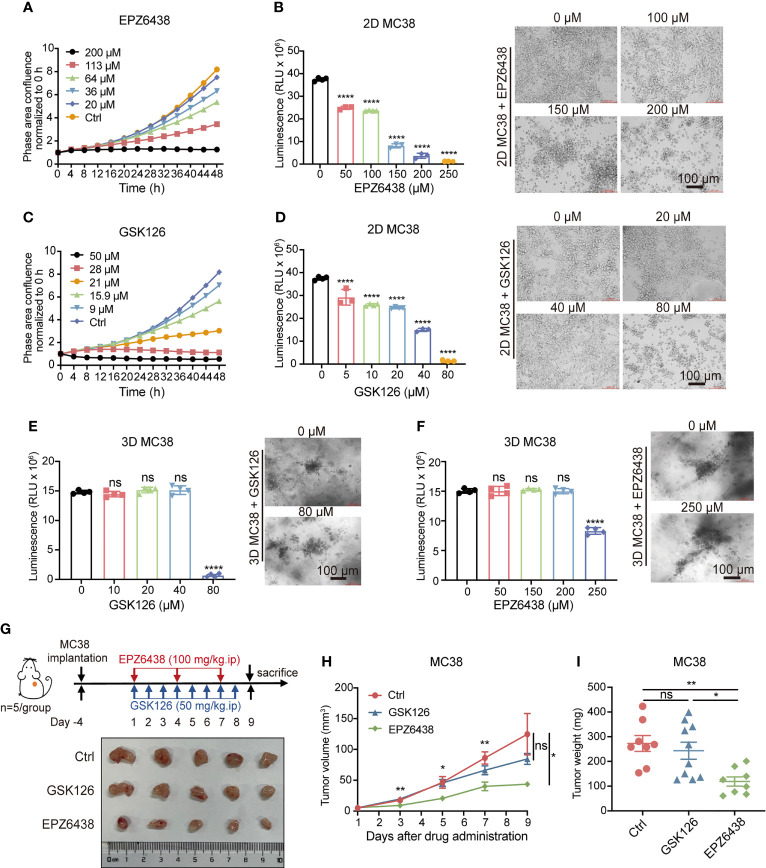
Inhibition of EZH2 by EPZ6438 had more of an effect on tumor growth than GSK126 in tumor-bearing mice with less cytotoxicity. **(A, C)** Effects of different concentrations of EPZ6438 **(A)** and GSK126 **(C)** on MC38 cell proliferation were detected every 4 h for 48 h. Concentrations are shown in different colors as indicated. **(B, D)** Cell viabilities were detected at different concentrations of EPZ6438 **(B)** and GSK126 **(D)** in MC38 2D cell lines. Representative images are shown on the right of the statistical graph. **(E, F)** 3D MC38 tumor spheroids were seeded in IBAC SR1 3D plates and grown for 6 days for spheroid formation and treated with indicated concentrations of EPZ6438 **(E)** and GSK126 **(F)** for 72 h after spheroid formation. Cell viabilities were detected at different concentrations of EPZ6438 **(E)** and GSK126 **(F)** in MC38 3D tumor spheroids. Representative images are shown on the right of the statistical graph. Scale bar = 100 µm. Three independent experiments were conducted. **(G)** Upper panel: schematic illustration of EZH2 inhibitor treatment schedule. Effect of GSK126 and EPZ6438 on the growth of MC38 cells in C57BL/6 mice. GSK126 was delivered daily and EPZ6438 twice a week until the end of the experiment. Lower panel: general tumor pictures of one of the independent experiments are shown after different treatments. **(H, I)** Statistical analysis of tumor volume **(E)** and tumor weight **(I)** are exhibited. Differences in tumor growth were assessed at the last time point. Mean ± SEM are shown. One-way ANOVA with Turkey’s multiple comparison post-test was used to evaluate statistical significance, (*p < 0.05, **p < 0.01, ****p < 0.0001, ns, no significance).

To verify the inhibitory effect of EPZ6438 and GSK126 on colorectal cancer, we first treated MC38 cell line with these two drugs respectively. The Incucyte^®^ live cell analysis system was used to monitor cell density reflected in cell proliferation with different concentrations of EPZ6428 (**Figure 1A**) and GSK126 (**Figure 1C**). We also detected cell viabilities of MC38 cells under EPZ6438 and GSK126 treatment, which showed that both EPZ6438 and GSK126 suppressed MC38 growth in a dose-dependent manner (**Figures 1B, D**). The IC50 value was calculated to be 85 μM for EPZ6438 and 20 μM for GSK126 (**Figure S1A**). We also established a 3D MC38 tumor spheroid model to better reflect the inhibitory effect of EZH2i on tumor growth. The results showed that the sensitivity of 3D cell spheres to EHZ2i was significantly decreased compared with 2D cells. However, high concentration of EHZ2i can still inhibit cell viability and reduce the tight connection of cell spheres (**Figures 1E, F**). In order to fully illustrate the inhibitory effect of EZH2i on colorectal cancer, we also used human CRC cell line RKO to repeat the above experiments (**Figure S1B–F**).

The data showed that the inhibitory effect of GSK126 on both 2D and 3D cell models was more obvious than EPZ6438, with lower drug inhibitory concentration and IC50 value. Given these observations *in vitro*, we wondered whether GSK126 would be more effective than EPZ6438 in *in vivo* experiments. Then we constructed a subcutaneous MC38 cell tumor-bearing mouse model and treated mice with 100 mg/kg of EPZ6438 every 3 days and 50 mg/kg GSK126 once a day intraperitoneally based on previous studies (23, 24) (**Figure 1G**). The result showed that EPZ6438 inhibited mouse tumor bearing but GSK126 had no significant inhibitory effect during the period of observation (**Figure 1H**). At the endpoint, mice were sacrificed and tumors were measured, which showed that tumor weight was decreased by EPZ6438 but not GSK126 (**Figure 1I**). These data suggest that EPZ6438 not only has more of an inhibitory effect on tumors *in vivo*, but also has less cytotoxicity compared with GSK126.

### EZH2 Inhibitors Affected the Cell Viability and Polarization of Macrophages

**Figure 2:**
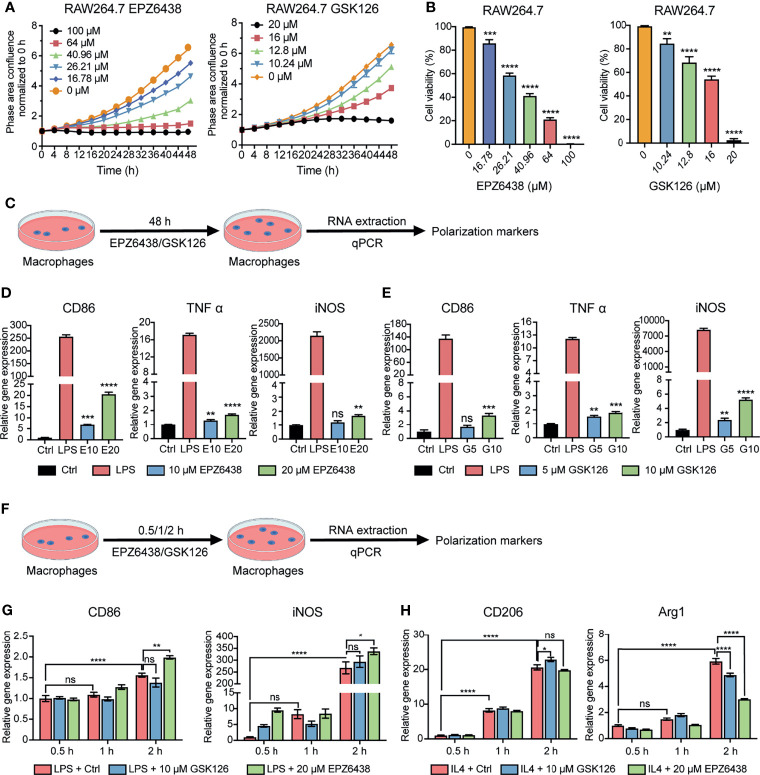
EZH2 inhibitors EPZ6438 and GSK126 induced M0 macrophages to differentiate into the M1 phenotype. **(A)** Effects of GSK126 and EPZ6438 on the proliferation in RAW264.7 cells every 4 h until the end of 48 h. **(B)** Cellular viability of RAW264.7 cells was detected after EPZ6438 and GSK126 treatment. **(C)** Schematic illustration of EZH2 inhibitor treatment schedule. **(D, E)** RAW264.7 cells were treated with EPZ-6438 (10, 20 μM) **(D)** and GSK126 (5, 10 μM) **(E)** for 48 h. Control cells were maintained in a medium supplemented with DMSO throughout the entire experimental period. An additional group of cells was treated with LPS (100 ng/ml) for the last 12 h. M1-type macrophage genes (*CD86, TNFα, and iNOS*) were analyzed by RT-PCR. **(F)** Schematic illustration of EZH2 inhibitor treatment schedule. EZH2 inhibitor and LPS (100 ng/ml) or IL-4 (20 ng/ml) were added into the culture medium of RAW264.7 cells at the same time. Effects of EPZ6438 and GSK126 on macrophage polarization were detected at time points of 0.5 h, 1 h, and 2 h, respectively. **(G, H)** M1-type macrophage genes (*CD86* and *iNOS*) **(G)** and M2-type macrophage genes (*CD206* and *Arg1*) **(H)** were analyzed by RT-PCR. Data are shown as mean ± SEM and three independent experiments were involved. *p < 0.05, **p < 0.01, ***p < 0.001, ****p < 0.0001, ns, no significance.

To investigate the effect of EZH2i on macrophage polarization, we first examined the effects of EZH2 inhibitors on proliferation and viability using macrophage cell line RAW264.7. We used the Incucyte^®^ live cell analysis system and CCK8 experiment to detect the effects of EPZ6438 and GSK126 at different concentration gradients on proliferation and viability of macrophages (**Figures 2A, B**). The data showed that both EPZ6438 and GSK126 also worked in a concentration-dependent manner. The toxicity of GSK126 on macrophages was stronger than that of EPZ6438, which was consistent with the results of *in vitro* experiments using tumor cell lines.

In order to explore the regulation of macrophage polarization and function at the concentration of EZH2i without affecting the survival of macrophages, we selected two concentrations of EZP63438 (10, 20 μM) and GSK126 (5, 10 μM) that slightly suppressed the cell viability of macrophages (**Figure 2C**). The results showed that both EPZ6438 and GSK126 could promote the polarization of M1 macrophages (*CD86*, *TNF-α*, and *iNOS*) (**Figures 2D, E**). However, we also observed that EZH2i treatment of macrophages for 48 h could also lead to increased expression of M2 polarization-related markers (*CD206*, *MRC1*, and *Arg1*) (**Figures S2A, B**). Therefore, to better observe the role of EZH2i in the procession of macrophage polarization, RAW264.7 cells were treated with both EZH2i as well as 100 ng/ml of LPS and 20 ng/ml of IL-4 simultaneously (**Figure 2F**). We collected RAW264.7 cells at different time points (0.5 h, 1 h, 2 h) after treatment for qPCR detection. The data showed that EPZ6438 can significantly promote the expression of *CD86* and *iNOS* to induce M1 macrophage polarization (**Figure 2G**). In the meantime, EPZ6438 significantly decreased the expression of M2 macrophage marker *Arg1* and tended to decrease *CD206* expression (**Figure 2H**). On the contrary, GSK126 showed a tendency to inhibit the polarization of M1 macrophages and promote the polarization of M2 macrophages (**Figures 2G, H**). Therefore, the above results suggested that EPZ6438 can promote M1-type macrophage polarization and inhibit M2-type polarization, and GSK126 had the reverse effect.

In addition, we also detected the expression of PD1/PD-L1 at the mRNA level during the polarization of M1/2 macrophages. The results showed that the expression of PD1/PD-L1 was significantly increased during the polarization of macrophages toward the M1 type (**Figure S2C**). However, in the process of polarization toward M2, the increase of the mRNA level of PD1/PD-L1 was not as significant as the change of M1 polarization (**Figure S2D**). Therefore, PD/PD-L1 expressed by macrophages may be related to their inflammatory states, which also correlates with their tumor-killing ability.

### EPZ6438 Promoted RAW264.7 Polarization Toward the M1 Phenotype in Tumor-Condition Medium

**Figure 3:**
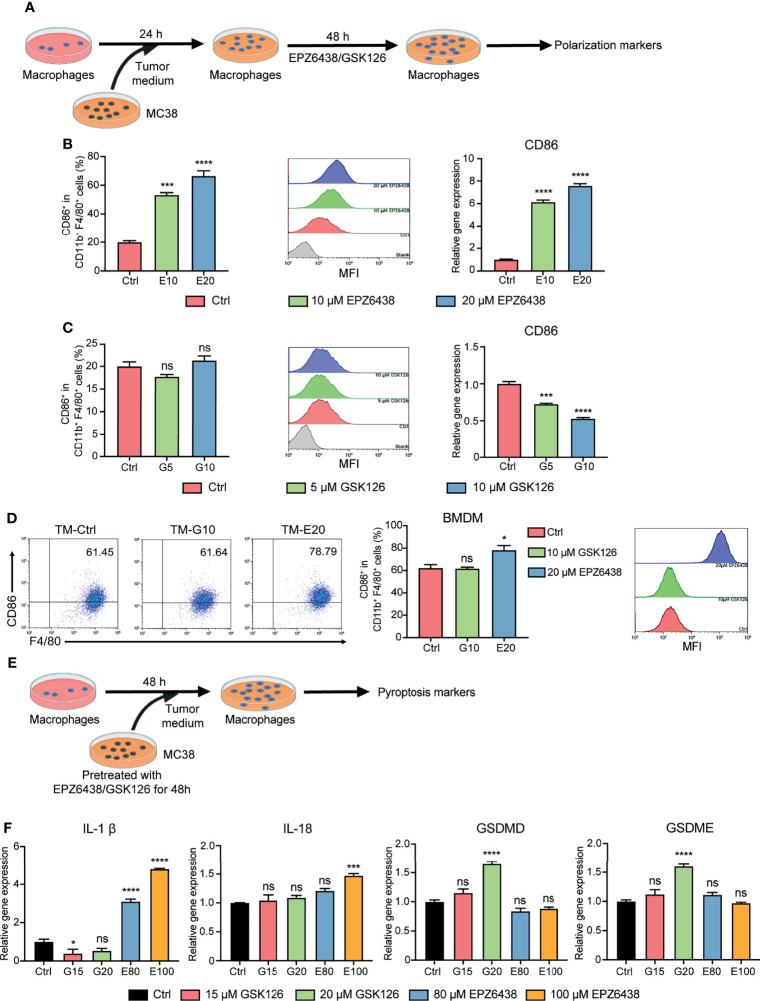
EPZ6438 and GSK126 induced M0-type RAW264.7 cells and BMDMs to differentiate into the M1 phenotype in the tumor microenvironment. **(A)** Schematic illustration of EZH2 inhibitor treatment with tumor-condition medium. **(B, C)** EPZ6438 (10, 20 μM) **(B)** or GSK126 (5, 10 μM) **(C)** were added to RAW264.7 cells pre-treated for 24 h with tumor-condition medium for another 48 h. Control cells were treated with DMSO. The statistical graph of flow cytometry on CD86^+^ macrophages ratio in CD11b^+^ F4/80^+^ (left), the histogram of mean fluorescence intensity of CD86^+^ cells (middle), and mRNA level of CD86 were analyzed by RT-PCR (right). **(D)** BMDMs were treated with 20 μM EPZ6438 and 10 μM GSK126 using the above treatment conditions. The scatter diagram of flow cytometry on CD86^+^ macrophages ratio in CD11b^+^ F4/80^+^ (left), the statistical graph of flow cytometry on CD86^+^ macrophages ratio in CD11b^+^ F4/80^+^ (middle), and the histogram of mean fluorescence intensity of CD86+ cells (right). **(E)** Schematic illustration of EZH2 inhibitor treatment with tumor-condition medium. **(F)** RAW264.7 cells were treated with tumor-condition medium, and MC38 cells were pre-treated with EPZ6438 (80, 100 μM) and GSK126 (15, 20 μM) for 48 h. Control cells were treated with the medium of MC38 cells with pre-treated DMSO. Pyroptosis-related genes (*IL-1β, IL-18, GSDMD, and GSDME*) were analyzed by RT-PCR. Data are shown as mean ± SEM and are representative of one out of three independent experiments with similar results. *p < 0.05, ***p < 0.001, ****p < 0.0001, ns, no significance.

To further investigate the tumor microenvironment function on macrophage polarization, we constructed a co-culture system of the supernatants of MC38 tumor cells with RAW264.7 macrophages. RAW264.7 cells were pre-treated with conditional medium of tumor cells for 24 h to mimic tumor-associated macrophages. Then, replacement fresh condition medium was added to EPZ6438 and GSK126 for another 48 h (**Figure 3A**). Flow cytometry was used to detect the effect of EZH2i on macrophage polarization (**Figure S3A**). The data showed that EPZ6438 could significantly increase the expression of CD86 in macrophages in the cell surface and total mRNA level of *CD86* (**Figure 3B**). But GSK126 treatment resulted in no significant change in CD86 mean fluorescence intensity, even that the mRNA level showed a decrease (**Figure 3C**). We also extracted mice bone marrow-derived macrophages (BMDMs) to performing the same experiment above. The results showed that CD86 mean fluorescence intensity (MFI) increased only under EPZ6438 treatment but not GSK126 (**Figure 3D**). The results showed that the outcome of experiments using primary macrophages from mice were consistent with others using the RAW264.7 cell line.

M1 macrophages release pro-inflammatory cytokines to play an anti-tumor role. Pyroptosis is a type of programmed cell inflammatory necrosis, which is characterized by increased expression of pro-inflammatory cytokines IL-1β, IL-18, gasdermin D (GSDMD), and gasdermin E (GSDME). Therefore, we further excluded the effect of pyroptosis on the inflammatory phenotype of macrophages. We first treated MC38 tumor cells with EPZ6438 (80, 100 μM) and GSK126 (15, 20 μM) to inhibit MC38 tumor growth for 48h and collected culture medium supernatant. Then, we used these conditional medium-treated RAW264.7 cells to detect whether the supernatant contained EHZ2i could induce pyroptosis in RAW264.7 cells (**Figure 3E**). The results showed that the GSK126 treatment group did not affect the release of inflammatory cytokines from macrophages, but it caused the increase of GSDMD and GSDME expression. On the contrary, the EPZ6438 treatment group improved the release of IL-1β and IL-18, but it did not affect the mRNA level of *GSDMD* and *GSDME* (**Figure 3F**). Therefore, these data suggest that EPZ6438 can not only exert tumor inhibition of MC38 cells, but also promotes RAW264.7 polarization to the M1 type in the tumor microenvironment of EPZ6438 treatment, without causing inflammatory necrosis of macrophages.

### EZH2 Inhibitors Reversed M2-Type Macrophage Polarization

**Figure 4:**
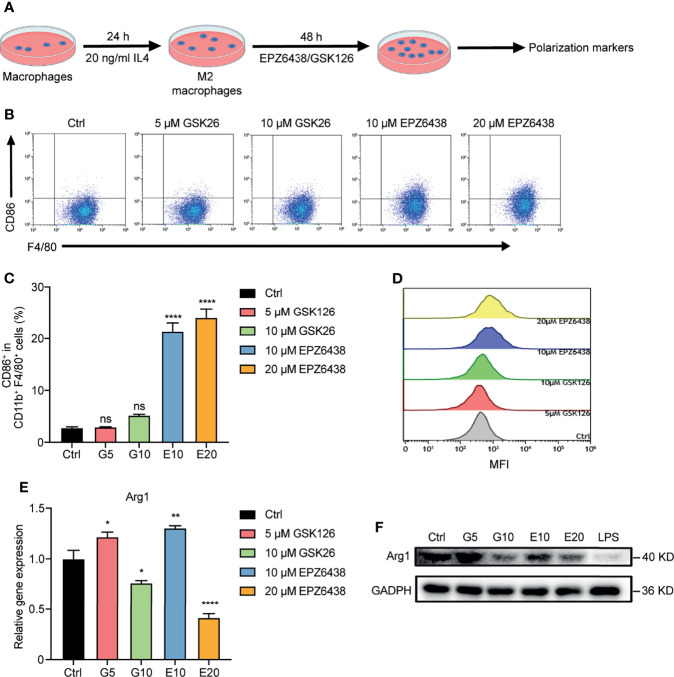
EPZ6438 reversed M2 macrophage re-polarization toward the M1 type. **(A)** Schematic illustration of EZH2 inhibitor treatment schedule. **(B)** RAW264.7 cells were pre-treated for 24 h with IL-4 (20 ng/ml) to induce M2 macrophages. Then, fresh medium was added with EPZ6438 (10, 20 μM) or GSK126 (5, 10 μM) and treated for another 48 h. Control cells were treated with DMSO. The scatter diagram of flow cytometry on CD86^+^ F4/80^+^ cells ratio. **(C)** The statistical graph of flow cytometry on CD86^+^ macrophages ratio in CD11b^+^ F4/80^+^ cells. **(D)** The histogram of mean fluorescence intensity of CD86^+^ cells. Gray color represents the control sample; red and green colors represent 5 μM and 10 μM GSK126, respectively; blue and yellow colors represent 10 μM and 20 μM EPZ6438, respectively. **(E)** mRNA level of *Arg1* was analyzed by RT-PCR according to the EZH2 inhibitor treatment schedule described in **(A)**. Data are shown as mean ± SEM and are representative of one out of three independent experiments with similar results. *p < 0.05, **p < 0.01, ****p < 0.0001, ns, no significance by one-way ANOVA. **(F)** The protein level of Arg1 was detected by Western blot. The protein level of Arg1 was significantly downregulated after different doses of GSK126 and EZP6438.

The macrophages could be changed to alternatively activated macrophages (M2 type) in the tumor microenvironment. Therefore, we pre-treated RAW264.7 with 20 ng/ml of IL-4 for 24 h to induce them into M2 macrophages firstly, and then treated them with EZH2i to observe whether EZH2i could reverse M2 macrophage polarization to the M1 phenotype (**Figure 4A**). The flow cytometry results showed that compared to GSK126 (5, 10 μM), EZP63438 (10, 20 μM) could significantly increase the expression of CD86, which reversed M2 macrophage polarization toward the M1 type (**Figures 4B–D**). To further verify whether EZH2i also inhibited M2 macrophage function, we detected changes in Arg1, an enzyme that represents M2 macrophage polarization. The data showed that both EZH2i could inhibit the protein and mRNA level of *Arg1* (**Figures 4E, F**). These results were consistent with the above observation of the effect of EZH2i on the procession of M2 polarization (**Figure 2H**). Therefore, these results suggest that EZH2 inhibitors, especially EPZ6438, can reverse the polarization of M2-type macrophages to M1-type macrophages.

### EPZ6438 Exerted Tumor Inhibition by Inhibiting the Proportion of M2-Type Macrophages in the Tumor Microenvironment *In Vivo*

**Figure 5:**
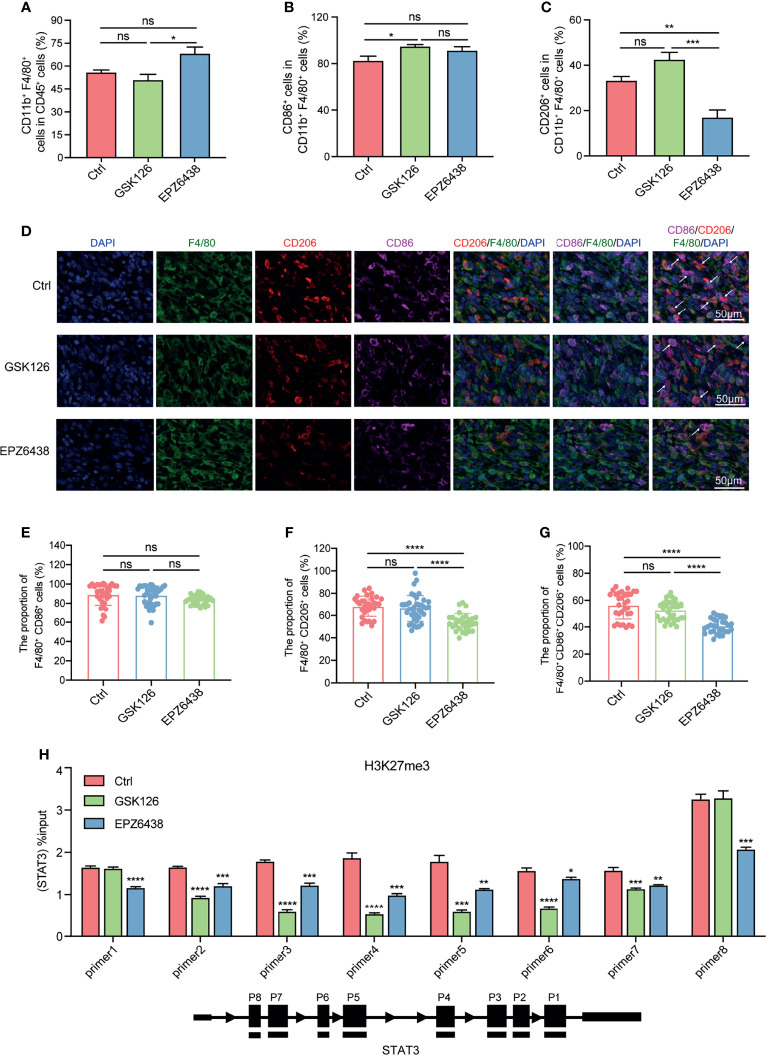
| EPZ6438 decreased the infiltration proportion of M2 macrophages but GSK126 increased it *in vivo*. **(A)** The statistical graph of flow cytometry on CD11b^+^ F4/80^+^ cell ratio in CD45^+^ cells representing total macrophages in the tumor microenvironment. **(B)** The ratio of CD86^+^ cells in CD11b^+^ F4/80^+^ cells representing M1 macrophages. **(C)** The ratio of CD206^+^ cells in CD11b^+^ F4/80^+^ cells representing M2 macrophages. **(D)** Opal multiplex immunofluorescence staining identified the proportion and location of M1 and M2 macrophages in the tumor microenvironment. Individual staining images of DAPI, F4/80, CD206, CD86, separately (left) and the merged images (right). **(E–G)** The quantitative analysis regarding the Opal multiplex immunofluorescence staining as shown above. **(H)** The level of H3K27me3 on the promoter of STAT3 was analyzed by the ChIP-qPCR experiment. The level of H3K27me3 was decreased after GSK126 and EPZ6438 treatment. The experimental results were normalized to a 2% input sample group. *p < 0.05, **p < 0.01, ***p < 0.001, ****p < 0.0001, ns, no significance.

Given these observations in the RAW264.7 cell line and BMDMs, we wondered whether EPZ6438 suppressed the proportion of M2-type macrophages in the tumor-bearing mouse model. As EPZ6438 had less cytotoxicity on macrophages and was more effective in regulating their polarization and function, we hypothesized that EPZ6438 exerts tumor inhibition *in vivo* partly by increasing the proportion of M1-type macrophages and decreasing the proportion of M2-type macrophages. Then, we dissociated tumor tissues from mouse models into single cells and analyzed the changes of macrophages in the tumor microenvironment by flow cytometry (**Figure S3B**). The data showed that EPZ6438 treatment had an increasing trend of infiltration of total macrophages compared with control and GSK126 groups (**Figure 5A**). For M1-type CD86^+^ cells, there were no statistically significant differences between the three treatment groups. Compared with the control group, the GSK126 treatment group showed an increasing trend (**Figure 5B**). However, for M2-type CD206^+^ macrophages, EPZ6438 significantly reduced their level, whereas the GSK126 group showed an increasing trend (**Figure 5C**). These results suggest that the reason why EPZ6438 has a more significant tumor suppressive effect than GSK126 is possibly by reducing the infiltration of M2-type macrophages.

In order to observe the number and location of CD86^+^ and CD206^+^ macrophages in the tumor microenvironment under GSK126 and EPZ6438 treatment, we performed multiplex immunofluorescence (mIF) assays to observe F4/80, CD86, and CD206 with the Opal staining technique (**Figure 5D**). The data showed that the proportion of CD206^+^ macrophages significantly reduced under EPZ6438 treatment but not GSK126 in the tumor microenvironment, which is consistent with flow cytometry results (**Figure 5F**). However, there was no significant difference in the proportion of CD86^+^ macrophages among the three groups (**Figure 5E**). In the meanwhile, we found that most of the macrophages in the control group and GSK126 treatment group co-expressed CD206 and CD86 simultaneously. We guess that these co-expressed CD206 and CD86 macrophages do not have an anti-tumor effect, and the proportion of these macrophages will also increase with the increase of tumor volume (**Figure 5G**). Immunohistochemical pictures also showed that the proportion of CD206^+^ macrophages was significantly reduced after EPZ6438 treatment compared with control and GSK126 groups (**Figure S3C**)

In addition, we wondered whether EPZ6438 and GSK126 play a regulatory role in macrophage polarization epigenetically through the function of histone methyltransferase on the STAT3 promoter. We collected RAW264.7 macrophages treated in a mimic tumor microenvironment for ChIP-qPCR detection. The results showed that both EPZ6438 and GSK126 could affect H3K27me3 levels in different promoter regions of STAT3 (**Figure 5H**). These results suggest that EPZ6438 and GSK126 regulate the polarization of macrophages through their functions of histone methyltransferase.

### The Anti-Tumor Effect of EPZ6438 Treatment Was Impaired After Macrophage Depletion

**Figure 6:**
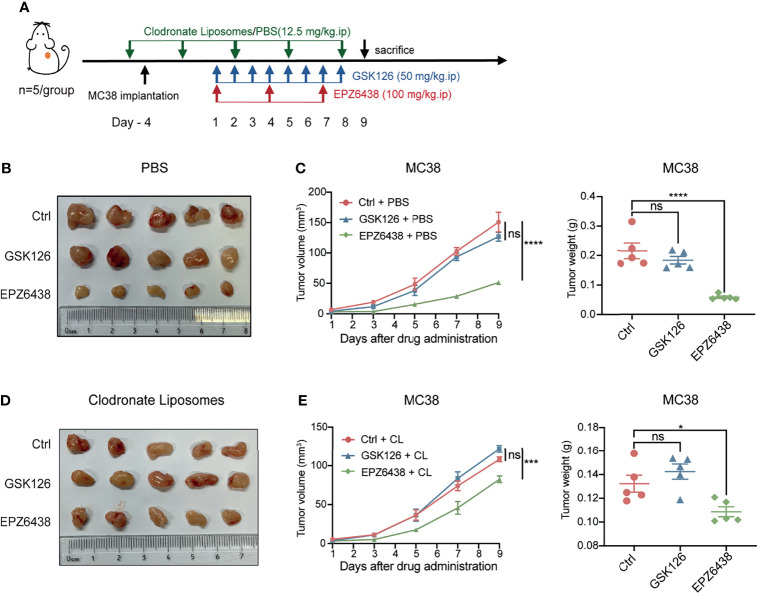
EPZ6438 played an anti-tumor role by regulating macrophages. **(A)** Schematic illustration of EZH2 inhibitor and clodronate liposome treatment schedule. **(B)** General tumor pictures of experiments are shown after EZH2 inhibitor and PBS control of clodronate liposome treatments. **(C)** Statistical analysis of tumor volume (left) and tumor weight (right) shown as **(B)** are exhibited. Differences in tumor growth were assessed at the last time point. **(D)** General tumor pictures of experiments are shown after EZH2 inhibitor and macrophage depletion of clodronate liposome treatments. **(E)** Statistical analysis of tumor volume (left) and tumor weight (right) shown as **(D)** are exhibited. Differences in tumor growth were assessed at the last time point. Mean ± SEM are shown. One-way ANOVA with Turkey’s multiple comparison post-test was used to evaluate statistical significance, *p < 0.05, ***p < 0.001, ****p < 0.0001, ns, no significance.

In order to further verify that EPZ6438 exerted a tumor inhibition effect by regulating the function of macrophages, we added a macrophage depletion treatment group used by CL and its control PBS to the animal experimental model. Mice were treated with EZH2 inhibitors combined with CL or PBS to observe how the combined application of macrophage depletion affected the anti-tumor function of EZH2 inhibitors. First, we evaluated the deplete efficiency of macrophages at 1 and 3 days by flow cytometry analysis. The data showed that the depletion effect of CL could last for 3 days (**Figure S4A**). Then, we treated the mice with CL or PBS 1 day before the implantation of tumors, and CL treatment was continued every 3 days during the experiment, as shown in **Figure 6A**. The data showed that EPZ6438 treatment still exerted a significant tumor inhibition effect in the PBS group compared with the placebo and GSK126 groups (**Figures 6B, C**). However, under the condition of CL treatment, the effect of tumor inhibition of EPZ6438 treatment was not obvious compared with PBS treatment. Although depletion of macrophages weakened its anti-tumor effect, EPZ6438 was still better than placebo and GSK126 (**Figures 6D, E**).

Moreover, we compared the effects of CL and PBS on the anti-tumor effect of drugs under different EZH2i treatments. The data showed that CL can inhibit tumor growth only by removing macrophages in mice (**Figure S4B**). These results not only indicated that macrophages were immune cells with abundant infiltration in colon cancer, but also indicated that tumor-associated macrophages can promote tumor growth. Moreover, macrophage clearance can slightly promote the anti-tumor effect of GSK126. Therefore, we considered that the cytotoxicity effects of GSK126 on immune cells are extensive. Even depletion tumor-associated macrophages, GSK126 cannot promote the tumor killing function of other immune cells (**Figure S4C**).

**Figure 7:**
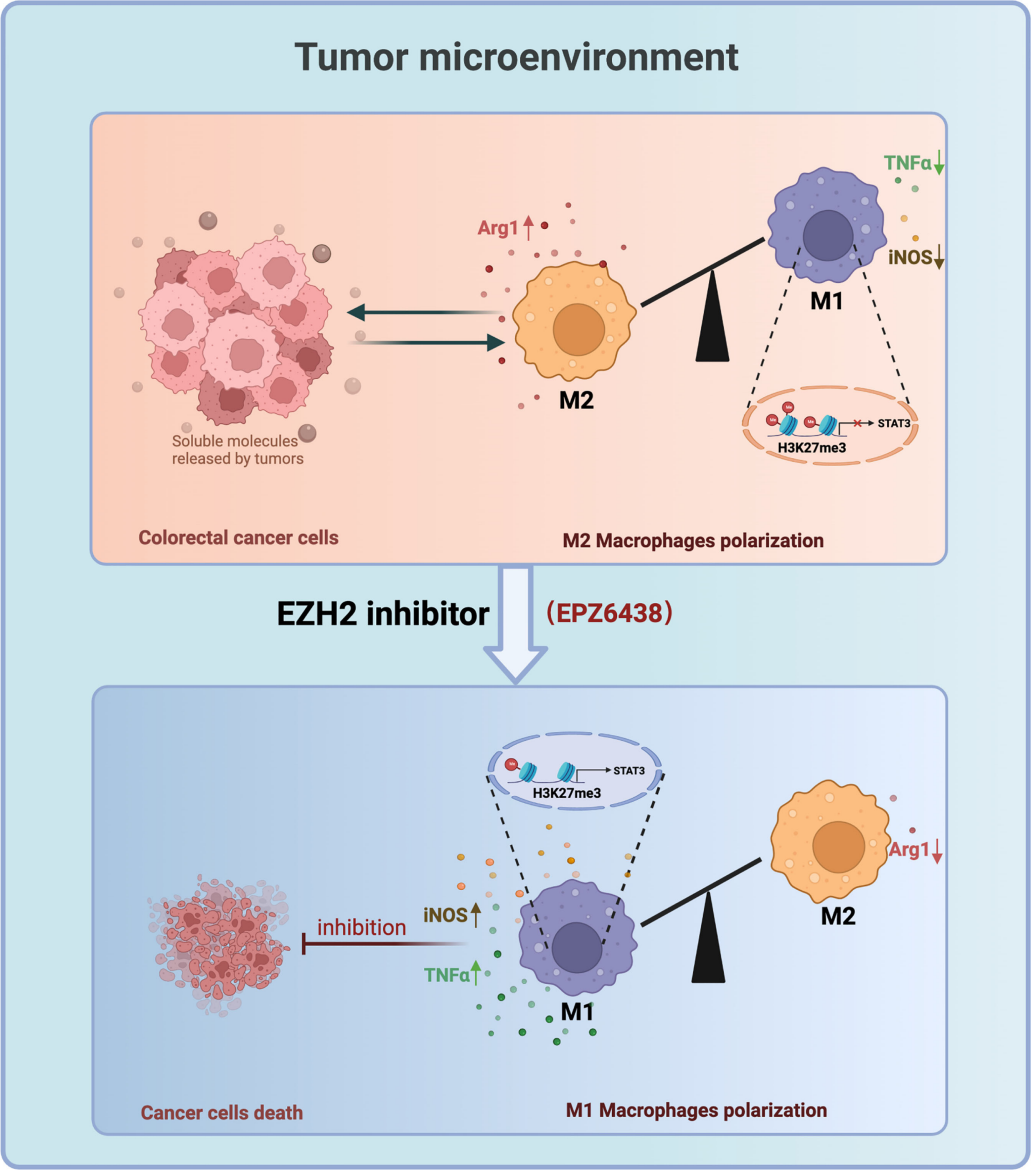
Working model: EZH2 Inhibitors Suppress Colorectal Cancer by Regulating Macrophage Polarization in the Tumor Microenvironment.

Taken together, our data suggested that EPZ6438 can regulate macrophages to exert suppression of colorectal tumors *via* decreasing the M2 macrophages and partly promoting the M1 macrophages in the tumor microenvironment. Mechanically, EZP6438 could reduce the H3K27me3 level on the promoter of STAT3 to induce upregulation of pro-inflammatory cytokines to exert anti-tumor effects (**Figure 7**). The reason why GSK126 could not inhibit tumor growth *in vivo* is partly because it cannot regulate macrophage polarization toward the tumor-killing type, which may be part of the reason why GSK126 did not work well in the clinical trial in solid tumors. Therefore, the therapeutic prospect of EPZ6438 is better than that of GSK126, which not only has less cytotoxicity *in vitro*, but also has better tumor suppression activity *in vivo*.

## Discussion

Besides the profound impact on tumorigenesis, EZH2 plays an essential role in immune regulation *via* trimethylating lysine 27 on histone H3 including macrophages. Although the EZH2 inhibitor was approved by the FDA in 2020, and has been applied in clinical practice, the benefit for patients suffering from solid tumors is unsatisfactory and several recent investigations have yielded contradictory results. Here, we found that inhibition of EZH2 with EPZ6438 and GSK126 suppressed CRC proliferation and growth in a model of 2D and 3D spheroids *in vitro*, but only EPZ6438 exerted a tumor inhibition effect on tumor-bearing mouse. Our data showed that anti-tumor effectiveness was at least partially related to the promotion of M1 polarization and the reduction of M2 polarization in the CRC tumor microenvironment. Mechanistically, we observed that pharmacological inhibition of EZH2 suppressed H3K27me3 modification on the promoter of STAT3, which activated macrophage polarization to the pro-inflammatory phenotype. Furthermore, the increasing of pro-inflammatory function of EPZ6438 was not induced by pyroptosis of macrophages using a co-culture system experiment *in vitro*. Thus, our data suggest that blocking EZH2 by EPZ6438, but not GSK126 suppresses CRC procession partially through decreasing M2-type macrophage proportion and increasing the M1 type in the TME, providing more evidence for EZH2 inhibitor use in CRC treatment.

CRC is one of the most frequent malignant tumors, ranks third in terms of morbidity and second in mortality worldwide, and results from an accumulation of both genetic and epigenetic alterations (25, 26). Disruption of histone methylation in CRC has drawn increasing interests in recent years (27). EZH2, as a histone methyltransferase, is increased significantly during the progression of CRC and associated with patient prognosis (2, 28, 29). Inhibition of EZH2 has been demonstrated to have promising therapeutic effects in preclinical CRC treatments (15, 30). Though lots of evidence has proved the therapeutic potential of targeting EZH2 in colon cancer, the effect of EZH2 inhibitors on anti-tumor immunity remains elusive. To better evaluate the responsiveness of CRC to EZH2 inhibitors, we established 3D cell spheroid models of MC38 and RKO cell lines. Our data indicated that GSK126 and EPZ6438 suppressed the proliferation of both CRC 2D and 3D cell models. Meanwhile, we further identified that EPZ6432 could reverse macrophage polarization induced by conditional tumor medium.

Macrophages are among the most common non-neoplastic cells in the CRC microenvironment, and their polarization state has prognostic significance in CRC (16, 31). EZH2, as an epigenetic regulator, plays an essential role in innate immune regulation including macrophages through regulation of differentiation, development, polarization migration, and so on (4, 32). Inhibition of EZH2 has been reported to be associated with enhanced NK and T-cell effector activities (33). Moreover, suppressing EZH2 activity by GSK126 increased myeloid-derived suppressor cells (MDSCs) and consequent suppression of anti-tumor immunity (6). Silvia et al. also reported that EPZ6438 enhanced monocyte recruitment and survival in multicellular spheroids (MCSs) (34). However, the regulation of EZH2 on macrophage polarization yielded controversial results in several investigations recently. Some studies suggested that macrophages tend to polarize toward the M2 type by inhibition of EZH2. For instance, Tang et al. reported that pharmacological inhibition of EZH2 ameliorated the indirect lung injury and inflammation post sepsis through blunting M1 macrophage polarization (35). They further confirmed that blockade of EZH2 with 3-DZNep not only alleviated the LPS-induced lung injury and inflammation through inducing M2 macrophages but also prevented against pulmonary fibrosis (36). They identified an obvious shift of macrophages from M1 to M2 subtypes in models of cecal ligation and puncture (CLP) mice and LPS-induced adult respiratory distress syndrome (ARDS). Moreover, Zhang et al. have documented that EZH2 deficiency suppressed M1 polarization and attenuated the inflammatory responses through the SOCS3/STAT1 pathway, leading to the suppression of autoimmune inflammation diseases including DSS-induced colitis in an experimental autoimmune encephalomyelitis (EAE) model (13).

However, in our study, we found that inhibition of EZH2 in the TME exhibited opposite effects, reflected by induced proinflammatory gene expression under the treatment of EPZ6438. This effect of EZH2 on macrophages is due to reducing the H3K27me3 level on the promoter of STAT3, a proinflammatory gene directly targeted by EZH2. These data are consistent with the study of Gareth et al. Their study found that EZH2 expression in macrophages can limit the activation of inflammatory response subjected to bacterial infection to restrict systemic spread of a localized infection (37). Furthermore, inhibition of EZH2 expression in glioma by siEZH2 or DZNep not only decreased the growth of glioma, but also delayed M2 macrophage polarization in a co-culture system of microglia and glioma cells and an *in vivo* experiment, which demonstrated the interaction between tumor cells and macrophages in the tumor microenvironment (5, 12). To systematically study the interaction between tumors and macrophages, we established an *in vitro* co-culture system of macrophages with tumor cells to simulate the tumor microenvironment. The involvement of the tumor microenvironment is one of the important reasons why our results are inconsistent with those results of the infectious and autoimmune disease models. Previous studies have demonstrated that suppressing EZH2 activity ameliorated experimental intestinal inflammation and delayed colitis-associated cancer progression (38). EZH2 expression was decreased in inflammatory bowel disease (IBD), and downregulation of EZH2 increased the expression of many inflammatory factors (39). In conclusion, EZH2 may play different regulatory roles in different disease models. In infection models and autoimmune disease models, EZH2 inhibits the expression of genes and signaling pathways involved in anti-inflammatory function, such as PPARγ, SOCS1, STAT6, etc. (35, 36). But in tumor models, EZH2 inhibits the expression of pro-inflammatory genes and pathways, such as SOCS3, STAT1, STAT3, etc. (5, 12, 39, 40). Specifically, the IL-6/STAT3 signaling pathway was also confirmed to be inhibited in M1-type macrophages but activated in M2-type macrophages in hepatocellular carcinoma (HCC) (41). Thus, it can be concluded that the regulation of EZH2 on M1/M2 polarization or pro/anti-inflammatory of macrophages depends at least on the different downstream target genes regulated by EZH2 in different disease environments.

EPZ6438 and GSK126 are two representative EZH2 inhibitors that have entered clinical trials and the former has been applied in clinical practice. In our study, the inhibition effect of these two inhibitors on tumor proliferation was shown to be inconsistent *in vivo* compared with *in vitro* experiments. At the drug tolerance dose explored in the previous experiment, EPZ6438 had better anti-tumor effects and was more safe than GSK126 in the mouse model. As mentioned before, at the beginning of 2020, EPZ6438 (tazemetostat) was approved for use in treatment of advanced or metastatic epithelioid sarcoma (18). Since then, clinical trials related to EPZ6438 have also increased, specifically, there have been 7 posted clinical trials from 2021, and so far a total of 33 clinical trials have been registered, including not only various solid tumors and hematological tumors, but also moderate or severe COVID-19 infection (NCT05018975). However, the clinical trial outcome of GSK126 released in 2019 was unsatisfactory in its phase I study, which indicated that the maximum-tolerated dose (MTD) of GSK126 had a relatively short half-life which limited effective exposure, and modest anti-cancer activity was observed at its tolerable doses (20). At present, that clinical trial on GSK126 has stopped, and a new GSK126-related clinical study has not been released. Previous studies have provided a potential mechanism behind the disappointing results of the phase I clinical trial of GSK126, suggesting that GSK126 resulted in increased numbers of myeloid-derived suppressor cells (MDSCs) and fewer effector T cells (6). Therefore, the further optimization of GSK126 in both dosage and re-structuring from a pharmaceutical chemistry aspect to overcome toxicity and extend the half-time *in vivo* is necessary. Furthermore, finding the proper combination such as combined with immunotherapies (such as anti-PD1, anti-PD-L1, and anti-CTLA4), targeting drugs, chemotherapies etc. may be the best approach to increase the anti-tumor effectiveness of GSK126 and other EZH2 inhibitors (42).

## Data Availability Statement

The raw data supporting the conclusions of this article will be made available by the authors, without undue reservation.

## Ethics Statement

The animal study was reviewed and approved by Peking University Third Hospital Medical Science Research Ethics Committee.

## Author Contributions

CL and JS contributed equally to this work. LX and YW conceived and designed the study. ZG, YG, XY, YL, and LC guided experimental methods. XM and YS guided the use of experimental equipment. TZ, JH, and RC collected the related paper. CL drafted the manuscript and JS, YW, and LX reviewed it. LX, YW, and CL were responsible for the final review of the manuscript. All authors read and approved the final manuscript.

## Funding

This study was supported by the National Natural Science Foundation of China (No. 81972966, and No.82001248) and Beijing Natural Science Foundation (No. 7214269).

## Conflict of Interest

The authors declare that the research was conducted in the absence of any commercial or financial relationships that could be construed as a potential conflict of interest.

## Publisher’s Note

All claims expressed in this article are solely those of the authors and do not necessarily represent those of their affiliated organizations, or those of the publisher, the editors and the reviewers. Any product that may be evaluated in this article, or claim that may be made by its manufacturer, is not guaranteed or endorsed by the publisher.

## Supplementary Material

Supplementary Figure 1 EZH2 inhibitors EPZ6438 and GSK126 also affected the proliferation and growth in human colorectal cancer cell line RKO. **(A, B)** IC50 values of EPZ6438 and GSK126 on MC38 **(A)** and RKO **(B)** cell lines. **(C, D)** Cell viabilities were detected at different concentrations of EPZ6438 **(C)** and GSK126 **(D)** in RKO 2D cell lines. Representative images are shown on the right of the statistical graph. **(E, F)** 3D RKO tumor spheroids were seeded in IBAC SR1 3D plates and grown for 6 days for spheroid formation and treated with indicated concentrations of EPZ6438 **(E)** and GSK126 **(F)** for 72 h after spheroid formation. Cell viabilities were detected at different concentrations of EPZ6438 **(E)** and GSK126 **(F)** in RKO 3D tumor spheroids. Representative images are shown on the right of the statistical graph. Scale bar = 100 µm. Three independent experiments were conducted.

Supplementary Figure 2 EZH2 inhibitors EPZ6438 and GSK126 induced M0 macrophages to differentiate into the M2 phenotype. **(A, B)** RAW264.7 cells were treated with EPZ-6438 (10, 20 μM) **(A)** and GSK126 (5, 10 μM) **(B)** for 48 h. Control cells were maintained in a medium supplemented with DMSO throughout the entire experimental period. An additional group of cells was treated with IL-4 (20 ng/ml) for the last 24 h. M2-type macrophage genes (*CD206, MRC1, and Arg1*) were analyzed by RT-PCR. **(C, D)** The mRNA levels of *PD1/PD-L1* were analyzed by RT-PCR during the process of macrophage polarization at different points of 0.5 h, 1 h, and 2 h.

Supplementary Figure 3 Flow diagram of flow cytometry analysis and immunohistochemical staining. **(A)** Flow diagram of flow cytometry analysis of .All the gates of flow diagrams were determined according to the position of blank samples. **(B)** Flow diagram of flow cytometry analysis of . **(C)** Immunohistochemical staining identified the expression of CD206+ M2 macrophages in the tumor microenvironment.

Supplementary Figure 4 Macrophage depletion by CL impaired the anti-tumor ability of EPZ6438. **(A)** Statistical graph of CL efficiency of macrophage depletion at the points of 1 and 3 days. **(B–D)** Changes in tumor size after depletion of macrophages by CL or control PBS in the placebo group **(B)**, GSK126 treatment group **(C)**, and EPZ6438 treatment group **(D)**. General tumor pictures (left), tumor volume (middle), and tumor weight (right).
