# Supplementary figures and images for: EZH2 Inhibitors Suppress Colorectal Cancer by Regulating Macrophage Polarization in the Tumor Microenvironment
Source: Front Immunol. 2022 Apr 1;13:857808. doi: 10.3389/fimmu.2022.857808 (PMC9010515; doi:10.3389/fimmu.2022.857808)

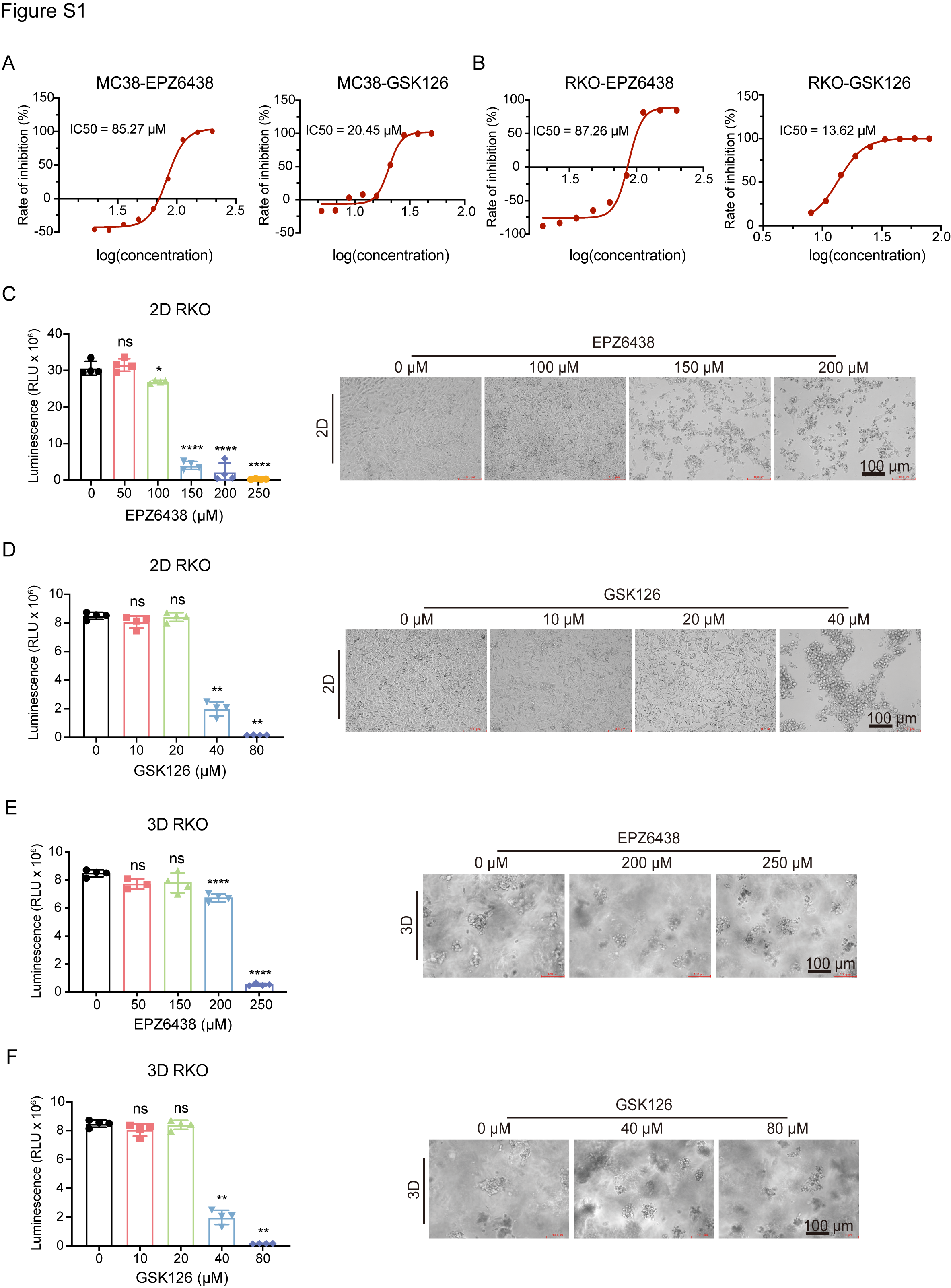

Supplement: Supplementary Figure 1 — EZH2 inhibitors EPZ6438 and GSK126 also affected the proliferation and growth in human colorectal cancer cell line RKO. (A, B) IC50 values of EPZ6438 and GSK126 on MC38 (A) and RKO (B) cell lines. (C, D) Cell viabilities were detected at different concentrations of EPZ6438 (C) and GSK126 (D) in RKO 2D cell lines. Representative images are shown on the right of the statistical graph. (E, F) 3D RKO tumor spheroids were seeded in IBAC SR1 3D plates and grown for 6 days for spheroid formation and treated with indicated concentrations of EPZ6438 (E) and GSK126 (F) for 72 h after spheroid formation. Cell viabilities were detected at different concentrations of EPZ6438 (E) and GSK126 (F) in RKO 3D tumor spheroids. Representative images are shown on the right of the statistical graph. Scale bar = 100 µm. Three independent experiments were conducted. [file Image_1.tif]

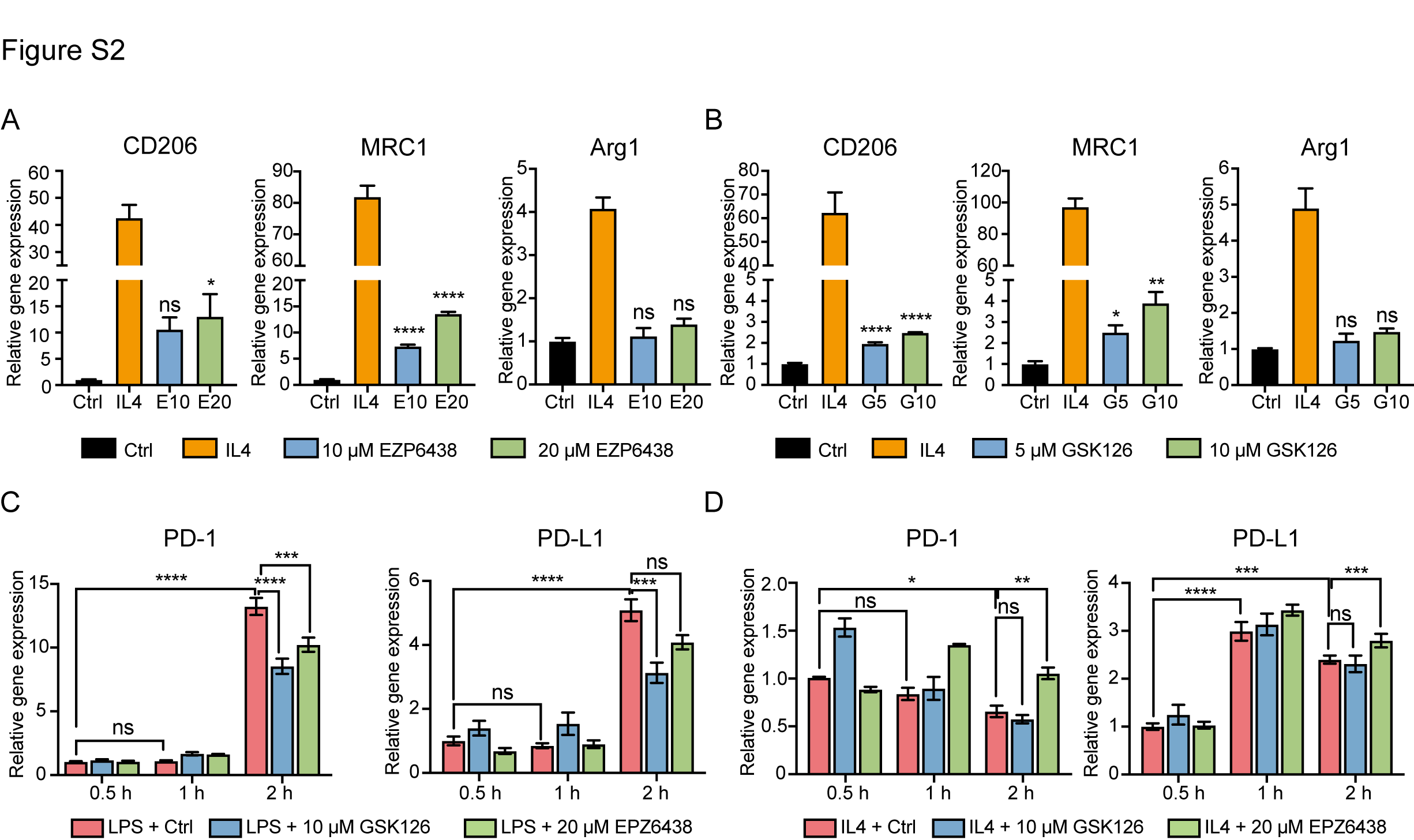

Supplement: Supplementary Figure 2 — EZH2 inhibitors EPZ6438 and GSK126 induced M0 macrophages to differentiate into the M2 phenotype. (A, B) RAW264.7 cells were treated with EPZ-6438 (10, 20 μM) (A) and GSK126 (5, 10 μM) (B) for 48 h. Control cells were maintained in a medium supplemented with DMSO throughout the entire experimental period. An additional group of cells was treated with IL-4 (20 ng/ml) for the last 24 h. M2-type macrophage genes (CD206, MRC1, and Arg1) were analyzed by RT-PCR. (C, D) The mRNA levels of PD1/PD-L1 were analyzed by RT-PCR during the process of macrophage polarization at different points of 0.5 h, 1 h, and 2 h. [file Image_2.tif]

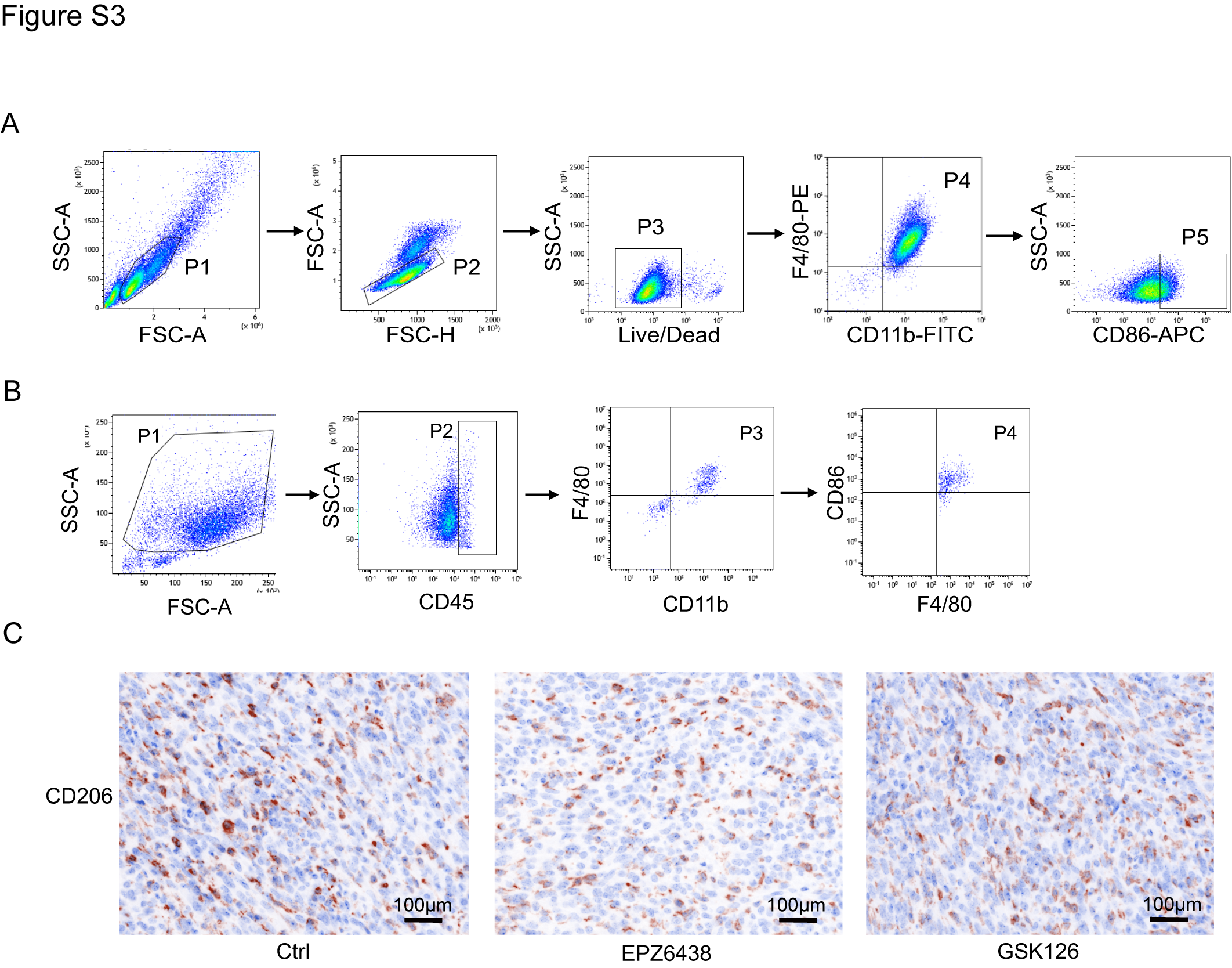

Supplement: Supplementary Figure 3 — Flow diagram of flow cytometry analysis and immunohistochemical staining. (A) Flow diagram of flow cytometry analysis of .All the gates of flow diagrams were determined according to the position of blank samples. (B) Flow diagram of flow cytometry analysis of . (C) Immunohistochemical staining identified the expression of CD206+ M2 macrophages in the tumor microenvironment. [file Image_3.tif]

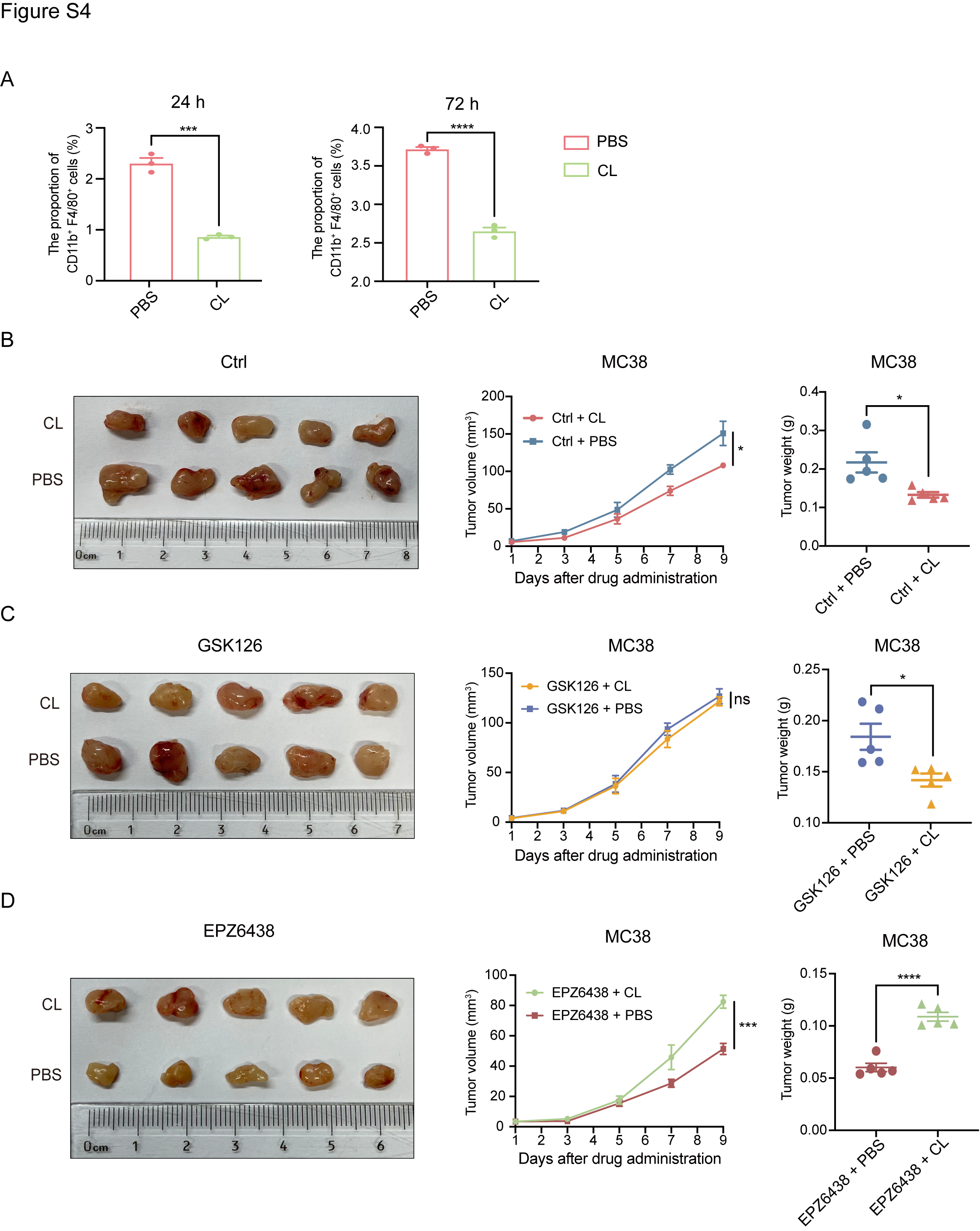

Supplement: Supplementary Figure 4 — Macrophage depletion by CL impaired the anti-tumor ability of EPZ6438. (A) Statistical graph of CL efficiency of macrophage depletion at the points of 1 and 3 days. (B–D) Changes in tumor size after depletion of macrophages by CL or control PBS in the placebo group (B), GSK126 treatment group (C), and EPZ6438 treatment group (D). General tumor pictures (left), tumor volume (middle), and tumor weight (right). [file Image_4.tif]
